# The Use of Stem Cells in Assisted Reproduction

**DOI:** 10.3390/jcm14196942

**Published:** 2025-09-30

**Authors:** Anna Szeliga, Anna Duszewska, Christian Unogu, Roman Smolarczyk, Stefania Bochynska, Gregory Bala, Blazej Meczekalski, Eli Y. Adashi

**Affiliations:** 1 Department of Gynecological Endocrinology, Poznan University of Medical Sciences, 60-179 Poznan, Poland; annamaria.szeliga@gmail.com (A.S.); christian.unogu@hotmail.com (C.U.); stefaniabochynska@gmail.com (S.B.); 2 Department of Morphological Sciences, Faculty of Veterinary Medicine, University of Life Science, 02-787 Warsaw, Poland; duszewskaanna@hotmail.com; 3 Department of Gynecological Endocrinology, Medical University of Warsaw, 02-097 Warsaw, Poland; rsmolarczyk@poczta.onet.pl; 4 School of Medicine, University College Dublin, D04 V1W8 Dublin, Ireland; greg.bala1@gmail.com; 5 Department of Medical Science, Brown University, Providence, RI 02903, USA; eli_adashi@brown.edu

**Keywords:** IVF, stem cells, assisted reproductive technology, ovarian rejuvenation, endometrial regeneration, sperm quality, ethics

## Abstract

**Background:** Infertility remains a significant global health challenge, affecting approximately 15% of couples worldwide. In vitro fertilization (IVF) has transformed reproductive medicine; however, challenges such as low success rates in older patients, ovarian insufficiency, endometrial dysfunction, and male infertility continue to limit outcomes. **Objective:** This review aims to summarize the principles of IVF and explore the potential role of stem cells in enhancing IVF outcomes, with particular attention to applications in both women and men, as well as the accompanying ethical considerations. **Summary:** Stem cell research has introduced novel therapeutic opportunities, including ovarian rejuvenation, endometrial regeneration, sperm quality enhancement, and the development of synthetic embryo models. Mesenchymal stem cells (MSCs), embryonic stem cells (ESCs), and induced pluripotent stem cells (iPSCs) demonstrate regenerative properties that may help to overcome current reproductive limitations. Despite encouraging findings from preclinical and early clinical studies, challenges such as tumorigenesis, genetic instability, and ethical controversies remain major barriers to translation. **Conclusions:** IVF continues to serve as a cornerstone of assisted reproductive technology (ART). Stem cell-based approaches represent an exciting frontier that could expand the therapeutic possibilities of IVF. Careful clinical validation, international regulatory harmonization, and robust ethical oversight will be essential to ensuring safe and equitable implementation.

## 1. Introduction

Infertility is a global health issue, affecting approximately 15% of couples worldwide, with significant medical, emotional, and socioeconomic consequences [1]. Since the first successful live birth following in vitro fertilization (IVF) in 1978 [1], assisted reproductive technology (ART) has become the cornerstone of infertility treatment. Despite major advances, IVF success remains limited by numerous factors, particularly reduced outcomes in older women, diminished ovarian reserve, endometrial dysfunction, and male factor infertility [2].

Globally, declining fertility rates and increasing maternal age at first childbirth further exacerbate these challenges. In women, age-related decline in oocyte quality and ovarian reserve significantly reduce IVF success rates [3], while uterine pathologies such as intrauterine adhesions impair implantation. In men, reduced sperm count, poor motility, and non-obstructive azoospermia (NOA) remain major causes of failed fertilization [4]. These persistent limitations highlight the urgent need for innovative therapies to complement conventional ART.

Stem cell-based approaches have emerged as a promising frontier in reproductive medicine. Stem cells, d efined by their self-renewal and differentiation potential, have already shown applications in regenerative medicine, organ repair, and disease modeling [5,6,7,8]. Recent studies suggest that they may enhance ovarian function, regenerate a damaged endometrium, and improve spermatogenesis [9,10,11,12]. Animal models and early clinical trials have provided encouraging data, although significant ethical, safety, and regulatory concerns remain [11,13,14].

The rationale for this review is to provide a comprehensive synthesis of the current state of knowledge regarding the role of stem cells in IVF. Specifically, we aim to:

1. Summarize IVF principles and persistent challenges;
2. Discuss the biological characteristics of stem cells and their mechanisms relevant to infertility;
3. Evaluate potential applications in women (ovarian rejuvenation, endometrial regeneration) and men (sperm quality enhancement);
4. Consider the ethical, clinical, and regulatory implications of stem cell-based ART.

By integrating these perspectives, this review seeks to bridge laboratory research and clinical practice, offering insights into how stem cell technologies may reshape the future of reproductive medicine.

## 2. IVF: Clinical Overview

In vitro fertilization (IVF) is a widely used assisted reproductive technology (ART) designed to help individuals and couples overcome infertility. Since the first successful birth in 1978, IVF has revolutionized reproductive medicine by enabling fertilization outside the human body, offering hope to millions worldwide who face difficulties conceiving naturally. This section provides an overview of the principles, procedures, indications, outcomes, risks, ethical considerations, and recent advances in the technology. IVF involves the retrieval of mature oocytes from a woman’s ovaries, their fertilization with sperm in a laboratory setting, and the subsequent transfer of one or more resulting embryos into the uterus to achieve pregnancy. This process bypasses physiological barriers that may prevent natural conception, such as tubal blockages or severe male factor infertility [1]. IVF is indicated for a variety of infertility causes, including tubal factor infertility, where blocked or damaged fallopian tubes prevent the egg and sperm from meeting [15]; male factor infertility, characterized by reduced sperm count, motility, or morphology [2]; endometriosis, where ectopic endometrial tissue negatively impacts fertility [3]; unexplained infertility, diagnosed when standard testing fails to reveal a definitive cause [4]; advanced maternal age, where declining ovarian reserve and oocyte quality contribute to reduced fertility [16]; and genetic disorders, where preimplantation genetic diagnosis (PGD) can be used to avoid disease transmission [17].

The IVF process consists of several key steps:

- Ovarian stimulation: Exogenous gonadotropins are administered to induce the development of multiple follicles, thereby increasing the number of retrievable oocytes [18]. Ultrasound and blood tests guide the timing of interventions.
- Oocyte retrieval: Mature oocytes are collected transvaginally under ultrasound guidance using a needle aspiration technique, typically performed under sedation [19].
- Fertilization: Retrieved oocytes are exposed to prepared sperm. Fertilization may occur via conventional insemination or through intracytoplasmic sperm injection (ICSI), in which a single sperm is injected directly into the oocyte, particularly in cases of severe male infertility [20].
- Embryo culture: Fertilized oocytes are cultured for 3–5 days in specialized media, allowing embryonic development. Embryo quality is assessed microscopically [21].
- Embryo transfer: One or more embryos are placed into the uterine cavity using a thin catheter inserted through the cervix. The number of embryos transferred depends on patient age, embryo quality, and guidelines designed to minimize multiple pregnancies [22].
- Luteal phase support and pregnancy test: Hormonal supplementation supports the endometrium, and pregnancy is confirmed approximately two weeks later via serum β-hCG measurement [23].

IVF success rates depend on several factors, including maternal age, ovarian reserve, embryo quality, and the underlying cause of infertility. Younger women (under 35 years) typically achieve live birth rates of 40–50% per cycle, whereas success rates decline with age [24]. The use of frozen embryo transfer has improved cumulative outcomes while reducing risks associated with fresh transfers [25]. Multiple pregnancies remain a major concern, given their association with maternal and neonatal complications. Accordingly, elective single embryo transfer (eSET) is increasingly recommended to balance efficacy with safety [26].

Although generally safe, IVF is associated with potential risks. These include ovarian hyperstimulation syndrome (OHSS), an exaggerated response to gonadotropins characterized by ovarian enlargement and fluid imbalance [27]; multiple pregnancies, which heighten risks of preterm birth, low birth weight, and maternal complications [28]; ectopic pregnancy, in which embryo implantation occurs outside the uterus [29]; and significant emotional, psychological, and financial stress [30].

IVF also raises important ethical considerations, such as the disposition of surplus embryos (freezing, donation, or destruction) [31], disparities in access to treatment [32], the potential misuse of IVF for non-medical sex selection or enhancement (“designer babies”) [33], and legal questions surrounding parentage and embryo custody [34]. National and international guidelines regulate IVF practice to ensure ethical standards and patient protection [35].

Recent advances continue to improve IVF outcomes. Time-lapse imaging enables continuous embryo monitoring and may enhance selection [36]. Preimplantation genetic testing for aneuploidy (PGT-A) improves implantation rates by identifying chromosomally normal embryos [37]. Cryopreservation techniques, such as vitrification, have increased embryo survival and clinical success rates [38]. Artificial intelligence (AI) is increasingly applied to embryo assessment [39]. Additionally, stem cell research is being explored for fertility enhancement, artificial gamete development, and better understanding of early embryogenesis [40].

**Figure 1:**
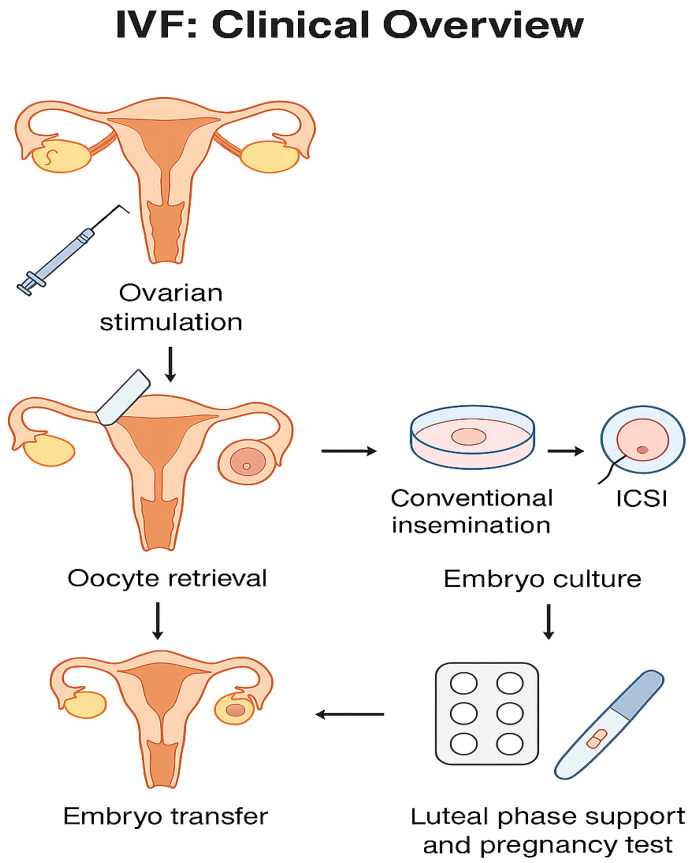
Overview of the IVF process, including ovarian stimulation, oocyte retrieval, fertilization (conventional insemination or ICSI), embryo culture, embryo transfer, and luteal phase support with pregnancy testing.

Figure 1 illustrates the sequential steps of the IVF process, from ovarian stimulation to pregnancy testing.

## 3. Stem Cells: Characteristics and Mechanisms

### 3.1. Overview of Stem Cell Characteristics

Stem cells are a unique population of cells defined by two fundamental properties: the capacity for self-renewal, enabling virtually unlimited division, and the ability to differentiate into diverse cell types. Broadly, stem cells are categorized as embryonic stem cells (ESCs), which are derived from the embryoblast of the blastocyst, and adult stem cells (ASCs), which can be collected from organisms during both prenatal and postnatal development [6,13].

Beyond their scientific importance, stem cells hold considerable clinical value in regenerative and transplant medicine, as well as in personalized and combination therapies. They have been widely investigated for the treatment of numerous diseases, degenerative conditions, and injuries; for modeling human pathologies; for drug discovery; and for both autologous and allogeneic therapies [7,8,13,41,42].

### 3.2. Types of Stem Cells

Over time, the potential application of stem cells in infertility treatment has also been recognized [9,10,11]. To appreciate their relevance in this context, it is important to outline their origin and developmental potential.

Following fertilization, the early embryo contains totipotent cells (totiESCs), meaning that each individual cell has the capacity to generate a complete organism, including embryonic and extra-embryonic tissues such as the fetal membranes. As the embryo progresses to the blastocyst stage, cellular differentiation occurs. The embryoblast and trophoblast arise, and cells lose totipotency, becoming pluripotent (pluriESCs). The trophoblast develops into fetal membranes, including the yolk sac, amnion, allantois, and chorion. The embryoblast gives rise to the primary germ layers (PGLs) and primordial germ cells (PGCs). The three germ layers then differentiate into specialized stem cell populations:

1. Ectodermal stem cells (ectoSCs);
2. Mesodermal stem cells (mesoSCs), which later include mesenchymal stem cells (MSCs) and muscle stem cells (muscleSCs);
3. Endodermal stem cells (endoSCs).

With further embryonic development, stem cells become progressively specialized, differentiating into unipotent, oligopotent, or multipotent lineages. During prenatal and postnatal life, virtually all tissues and organs retain a pool of resident adult stem cells (ASCs, also termed somatic stem cells), which play a crucial role in tissue repair, regeneration, and homeostasis. Importantly, this pool diminishes with age [6,13,43].

### 3.3. Potential Applications of ESCs and iPSCs in Infertility and IVF

In laboratory settings, ESCs are isolated from the embryoblast of the blastocyst and are therefore pluripotent (pluriESCs). Although theoretically possible, isolating ESCs at earlier developmental stages would yield totipotent cells (totiESCs). In human research, however, ESC isolation is legally restricted to embryos less than 14 days post-fertilization [6,13].

By contrast, somatic stem cells can be isolated from prenatal or postnatal tissues, although this process is considerably more involved. These somatic cells (e.g., skin fibroblasts, adipocytes, or bone marrow-derived cells) can be reprogrammed into stem-like cells, termed induced pluripotent stem cells (iPSCs). Additionally, iPSCs may be derived from ESCs. Finally, iPSCs can be differentiated into any cell type [42,44,45].

Both ESCs and iPSCs possess self-renewal and differentiation potential. However, iPSCs are not identical to ESCs, as the reprogramming process can introduce mutations, tumorigenic risks, and immunogenicity. During the reprogramming of somatic cells into iPSCs, genomic and epigenetic changes may affect their stability and functionality. iPSCs may retain the epigenetic memory of their somatic parent cells, which may affect their differentiation potential. In addition, the differentiation of iPSCs into any cell type may also cause genetic instability, activating the cell’s tumorigenic potential. Genomic instability and abnormal gene expression of iPSCs may also lead to the rejection of differentiated cell transplants, even if derived from autologous iPSCs. Ethical controversies surrounding ESC isolation, which involves the destruction of embryos, further limit their use. Consequently, iPSCs are often considered the most promising source of pluripotent cells, as their generation is independent of gender, age, or embryonic origin [42,46].

In both ESCs and iPSCs, additional opportunities exist for gene editing (e.g., CRISPR-Cas9 technologies), artificial chromosome introduction, diagnosis of monogenic and chromosomal aberrations, predictive polygenic testing, sex selection, and micromanipulation techniques. These methods substantially broaden the potential of these cells in research and clinical applications [47,48,49,50].

The possibilities and prospects for the use of iPSCs and ESCs in humans in the context of infertility and ART/IVF can be classified in three pathways: (A) stem cell-based therapy in infertility, (B) in vitro gametogenesis (IVG), and (C) synthetic embryo models (SEMs). Most of these pathways remain in the early experimental stage, and only the first one has been clinically applied to humans. The other two are in the experimental phase, mainly based on laboratory models and animal studies [51].

### 3.4. Pathway A—Stem Cell-Based Therapy in Infertility

iPSCs, and to a lesser extent ESCs, have been investigated for cell- and tissue-based therapies in both female and male infertility. Clinical applications include the use of stem cells derived from adipose tissue (ADSCs), amniotic fluid (AFSCs), bone marrow (BMSCs), menstrual blood (MenSCs), mesenchymal stem cells (MSCs), umbilical cord (UCSCs), and Wharton’s jelly (WJMSCs). Therapeutic strategies may involve direct transplantation of stem cells or administration of their paracrine factors into reproductive tissues [9,11,12,52,53,54,55,56,57,58,59].

Although the concept of constructing artificial reproductive organs remains preliminary, initial reports from animal studies suggest that this may be possible [60,61]. Emerging evidence also points to early progress in reconstructing female and male reproductive structures in humans [58,62,63,64,65].

### 3.5. Pathway B—In Vitro Gametogenesis (IVG)

One of the most promising directions is in vitro gametogenesis (IVG). In this approach, oocytes and spermatozoa are derived from PGCs generated from ESCs or iPSCs. In females, PGCs undergo oogenesis, developing into oogonia and subsequently into oocyte stages I and II. In males, PGCs differentiate through the sequential stages of spermatogenesis, including gonocytes, spermatogonia, spermatocytes I and II, spermatids, and finally spermatozoa [5,10,66,67,68].

To date, no human procedure has successfully generated functional gametes from stem cells, despite extensive global research [69]. Mice remain the only species in which both oogenesis and spermatogenesis have been reconstructed in vitro from iPSCs. Importantly, these processes required co-culture with somatic cells from fetal gonads [46]. While oogenesis in mice resulted in viable offspring after ICSI, male differentiation produced only spermatids, which also required somatic support for maturation [70].

Problems in obtaining human gametes from iPSCs may result from the specificity of gametogenesis (oogenesis and spermatogenesis) and gonadogenesis, which affects the production of functional oocytes and spermatozoa. Many critical processes have been studied (e.g., demethylation, methylation, crossing over, and meiosis). However, cytoplasmic maturation of oocytes (including storage of lipids, proteins, sugar, and mRNA), interaction between the oocyte and granulosa cells, and spermiogenesis (including Golgi, cap, tail, and maturation phases) should also be studied.

Animal studies, particularly in livestock, provide valuable insights into gametogenesis models that more closely approximate human reproductive processes [51].

Beyond generating gametes for IVF, IVG offers potential for creating large banks of oocytes and sperm. Theoretically, any somatic cell could be deposited in such a bank, later reprogrammed into a stem cell, and ultimately differentiated into gametes.

### 3.6. Pathway C—Synthetic Embryo Models (SEMs)

Another frontier is the creation of synthetic embryo models (SEMs) through the self-organizing aggregation of ESCs or iPSCs [13,70,71,72,73,74]. These models mimic early embryonic development only up to the 14-day limit [13] and are primarily research tools. Notably, recent progress has demonstrated the production of PGCs from SEMs, thereby establishing a pathway toward gametogenesis [13].

Although SEMs are not currently totipotent, this may eventually be achievable. In theory, totipotent synthetic embryos (totiSEs) could be created, capable of developing fetal membranes, implanting, and progressing through post-implantation stages, ultimately resulting in live offspring. The possibility of creating monozygotic twins through microsurgical division of such embryos further illustrates the transformative but ethically challenging nature of this research.

## 4. Potential Applications in Women: Ovarian Rejuvenation and Endometrial Regeneration

Stem cells (SCs), characterized by their undifferentiated nature, self-renewal capacity, and potential to differentiate into specialized cell types, have emerged as a promising therapeutic strategy for ovarian insufficiency. Stem cell-based therapy offers the possibility of restoring impaired ovarian endocrine function, promoting tissue repair, and potentially recovering fertility in affected patients [73].

Previous studies have investigated mesenchymal stem cells (MSCs), embryonic stem cells (ESCs), induced pluripotent stem cells (iPSCs), and germline stem cells (GSCs) for ovarian dysfunction. Compared with conventional approaches to managing primary ovarian insufficiency (POI), stem cell transplantation provides several advantages: a favorable safety profile, minimal systemic toxicity, and the ability to restore ovarian function directly. As such, stem cell therapy is considered a promising avenue that addresses POI at its root cause and has become a major focus of current research [74,75].

Recent evidence indicates that stem cell transplantation can enhance ovarian function by upregulating key hormone expression and inhibiting granulosa cell apoptosis. Therapeutic efficacy in such cases can be evaluated using a variety of parameters, including folliculogenesis, granulosa cell apoptosis, angiogenesis, pregnancy outcomes, and modulation of hormone levels [76].

A key process in ovarian restoration is cell homing, whereby circulating or transplanted stem cells migrate selectively to target tissues. For example, intravenously administered hematopoietic stem cells and progenitors accumulate in bone marrow. Although the mechanisms underlying MSC homing remain incompletely understood, this property likely improves transplantation outcomes by reducing the number of cells required for effective engraftment [73].

The ability of MSCs or ovarian stem cells to differentiate into functional oocytes in cases of ovarian insufficiency remains uncertain. However, animal models suggest that MSCs may differentiate into granulosa cells, thereby restoring endocrine function and promoting folliculogenesis [77,78]. Furthermore, mitochondrial transfer from autologous adipose tissue-derived stem cells has been shown to improve oocyte quality, embryo development, and fertility in aged mice, offering a potential strategy for treating infertility in older women [79].

Beyond differentiation, the paracrine activity of stem cells is increasingly recognized as one of their most important therapeutic mechanisms in regenerative medicine. By secreting cytokines and growth factors, stem cells promote neovascularization, suppress inflammation, prevent apoptosis, reduce fibrosis, and regulate immune responses. In POI models, conditioned media from stem cells have been shown to protect ovarian tissue from age-related damage, underscoring the importance of paracrine signaling in enhancing ovarian function [80,81].

An additional strategy in the prevention of ovarian failure involves preventing granulosa cell (GC) apoptosis. Human placental mesenchymal stem cells (hPMSCs) have demonstrated protective effects by suppressing GC apoptosis and modulating stress responses through the IRE1α pathway. A more recent approach utilizes menstrual blood-derived stem cells (MenSCs), which are particularly attractive due to their non-invasive collection and regenerative potential [82,83].

Stem cells are also being explored for endometrial regeneration. Animal studies show that MSC transplantation reduces endometrial fibrosis, increases engometrial gland numbers, and enhances vascular regeneration. These findings have progressed to early clinical trials for intrauterine adhesion (IUA). Phase I trials indicate that MSC therapy can repair uterine damage, improve pregnancy rates, and demonstrate an excellent safety profile without severe adverse effects [59,84,85,86].

A recent meta-analysis confirmed that transplantation of stem cell-loaded scaffolds promotes endometrial regeneration. Reported benefits include increased endometrial thickness, greater gland density, reduced fibrosis, and improved implantation rates. Stem cell-loaded scaffolds generally improved the number of gestational sacs or implanted embryos, and treatment demonstrated anti-inflammatory, pro-angiogenic, and anti-apoptotic effects, although significant heterogeneity was observed across studies. Subgroup analyses suggested that outcomes varied across animal models; however, small sample sizes limited the strength of these conclusions [87]. Ovarian rejuvenation and endometrial regeneration are presented in the Table 1 in simplified form of clinical human studies.

## 5. Potential Applications in Men: Sperm Quality Enhancement

Infertility affects up to 30% of men, with common causes including low sperm count, poor motility, and poor sperm quality [97]. These challenges have prompted the search for innovative approaches within assisted reproductive technology (ART), particularly in vitro fertilization (IVF). Stem cell-based therapy represents one of the promising strategies for male infertility, especially in cases of impaired sperm production [98]. By enhancing sperm quality, such interventions may improve the success rates of IVF [45]. The stem cell populations currently under intense investigation include spermatogonial stem cells (SSCs), mesenchymal stem cells (MSCs), and induced pluripotent stem cells (iPSCs).

Spermatogonial stem cells (SSCs) are resident testicular stem cells that sustain sperm production throughout post-pubertal life [99]. Dysfunction or depletion of SSCs is a primary cause of non-obstructive azoospermia (NOA), a condition where sperm is absent from the ejaculate due to impaired spermatogenesis [99]. SSC transplantation is being investigated in clinical trials, particularly in men who have undergone gonadotoxic therapies, with the aim of restoring spermatogenesis and fertility [100,101]. Researchers are also working to culture and differentiate SSCs in vitro to produce mature sperm for IVF [102]. Remaining challenges include optimizing cryopreservation methods, avoiding contamination by malignant cells, and addressing sociocultural considerations [103].

Mesenchymal stem cells (MSCs) are also being explored due to their regenerative and immunomodulatory properties [22,52]. Within the testes, MSCs can establish a supportive microenvironment by secreting growth factors and cytokines that enhance germ cell survival, proliferation, and differentiation [23]. These effects may improve testicular function and spermatogenesis [44]. Systematic reviews and preclinical studies have demonstrated that MSC therapy reduces inflammation, promotes blood flow through angiogenesis, and repairs damaged reproductive tissue, thereby fostering conditions conducive to sperm production [24]. MSCs are particularly relevant in the context of azoospermia, including NOA [26]. Animal models have shown that MSCs can differentiate into germ-like cells and restore spermatogenesis, improving testicular histology and sperm production. Although these preclinical studies yield encouraging results, the small-scale human trials that have been undertaken lack standardized protocols and long-term safety data [27,28,29]. 

Induced pluripotent stem cells (iPSCs) have opened new possibilities for treating severe male infertility. By reprogramming adult somatic cells to an embryonic-like pluripotent state, iPSCs can differentiate into a wide range of cell types [30,42]. For men entirely lacking functional germ cells, this approach offers the potential to generate patient-specific sperm in vitro. Preclinical studies have successfully differentiated iPSCs into immature sperm-like cells, making an important step toward producing functional gametes for IVF [31,32,33]. The ultimate goal is to generate mature, genetically intact, and functional spermatozoa from patient-derived iPSCs, enabling men with severe infertility to father biologically related children. However, significant challenges remain, including ensuring accurate differentiation, preventing genetic abnormalities, and minimizing tumorigenic risks [34,35].

The integration of SSC-, MSC-, and iPSC-based therapies into mainstream IVF practice will depend on continued research to refine differentiation protocols, ensure reproducibility, and establish long-term safety. Ethical considerations, including genetic manipulation and equitable access, must also be addressed [36]. Nonetheless, stem cell-based strategies have the potential to transform male infertility management, significantly improving sperm quality and fertility outcomes, and positioning stem cells as a cornerstone of future IVF treatments.

Previous clinical studies of Potential Applications in Men: Sperm Quality Enhancement are presented in the Table 2.

## 6. Ethical Considerations of Stem Cell IVF

Global fertility decline has been documented for several decades, with the World Health Organization highlighting genetics, epigenetics, and environmental factors as contributing causes [100,101,102,103]. These concerns have fueled interest in stem cell-based interventions as potential solutions to safeguard reproductive capacity and, by extension, the continuity of the human species. While the application of stem cells to infertility treatment represents a major scientific advance, it also raises significant scientific, legislative, clinical, ethical, and social dilemmas [88,104,105,106,107,108]. Existing regulations, though important, require continual reassessment in light of ongoing scientific progress [9].

The primary ethical debate centers on the source of stem cells and their intended use. iPSCs may be derived from virtually any somatic cell collected during prenatal or postnatal life. In principle, they could be obtained from young infertile couples as part of treatment, which is widely accepted. However, concerns arise regarding the collection of cells from fetuses, older adults, deceased individuals, celebrities, or members of vulnerable populations, including LGBTQ+ and other individuals. The possibility of obtaining cells without proper consent, or worse even without the donor’s knowledge, raises fears of “cell kidnapping” [88,105,106,107,108].

Applications of iPSCs and, less frequently, ESCs in cell or tissue therapies (Pathway A) are relatively less controversial. For instance, their use in creating or repairing reproductive tissues or even entire reproductive systems is often considered ethically acceptable when appropriate safeguards are in place [12,107]. Nonetheless, specific issues, such as equitable access and long-term monitoring, require careful attention.

By contrast, in vitro gametogenesis (IVG, Pathway B) raises deeper ethical and legal dilemmas. IVG makes it theoretically possible for same-sex couples to have genetically related children, for a single individual to provide both gametes, and for embryos to be generated from multiple parents [12,105]. These possibilities challenge traditional notions of parenthood, kinship, and family law.

The most contentious area is the creation of synthetic embryos (SEs, Pathway C). Scientifically, the status of SEs remains debated. Authors have argued that they should not be considered true embryos [27,36], while others warn that blurring this distinction risks undermining ethical boundaries. If science itself struggles to define SEs, the ethical implications become even more complex. SEs could, in theory, enable implantation and post-implantation development, raising the prospect of entirely novel forms of reproduction. Many scholars caution that this could push assisted reproduction onto a profoundly dangerous pathway.

Together, these pathways highlight a fundamental shift in how human reproduction may be conceptualized. Sexual reproduction could be reduced to the combination of artificial gametes or even to the generation of synthetic embryos, potentially gestated in artificial wombs. In this scenario, conception becomes increasingly detached from the sexual act. This raises a critical question: does this represent genuine progress in the treatment of infertility, or does it risk becoming a technological substitution for procreation itself?

## 7. Challenges and Future Perspectives

Although stem cell-based technologies hold great promise for IVF, several major challenges must be addressed before they can be widely adopted in clinical practice. Key concerns include safety risks such as genetic mutations, tumorigenesis, and immunogenicity. Standardized and reproducible laboratory protocols remain lacking, and this variability hinders the translation of promising preclinical findings into routine use.

Another significant barrier is regulatory uncertainty. The status of artificial gametes, embryo models, and other novel technologies is not yet clearly defined in many jurisdictions, creating ambiguity for both researchers and clinicians. Ethical issues also loom large, including potential inequalities in access, and the redefinition of parenthood in cases involving artificial gametes or synthetic embryos [7,14,47,50].

To overcome these challenges, translational research must be expanded, with carefully designed preclinical and clinical studies ensuring long-term safety and efficacy. International collaboration will be essential to harmonize regulatory frameworks, establish clear ethical guidelines, and promote equitable access. Only through such coordinated efforts can stem cell-based IVF technologies advance responsibly toward clinical implementation.

## 8. Conclusions

IVF remains the cornerstone of infertility treatment, offering millions of individuals and couples the possibility of biological parenthood. Stem cell-based approaches represent an exciting new frontier with the potential to overcome current limitations by enabling ovarian rejuvenation, endometrial repair, and sperm quality enhancement. These applications highlight the regenerative potential of stem cells and their capacity to expand the therapeutic scope of assisted reproductive technology.

Nonetheless, ethical, regulatory, and scientific challenges remain central to clinical translation. The safe and equitable adoption of these technologies will depend on robust science, strong ethical oversight, and international cooperation. If these conditions are met, stem cell-assisted IVF could transform reproductive medicine, reshaping not only infertility care but also our broader understanding of human reproduction.

## Figures and Tables

**Table 1:** Clinical human studies on stem cell-based ovarian rejuvenation and endometrial regeneration.

First Author (Year)	Indication	Study Design	Type of Cells	Primary Endpoints	Reproductive and Overal Outcomes
Herraiz S. (2018) [88]	POR	Prospective pilot; *n* = 17	Autologous bone marrow-derived stem cells (BMDSCs);	Increase in AMH (some patients), increase in AFC, increase in the number of stimulable antral follicles and oocytes	Improved follicle and oocyte quantities, enabling pregnancy in women who were poor responders and previously limited to oocyte donation.
Igboeli P. (2020) [89]	POI	Two cases of Caucasian women; *n* = 2	Autologous bone marrow-derived stem cell engraftment into the ovary	Increase of 50% in the volume of treated ovaries, serum levels of estrogen increased by 150% compared with preoperative levels	Resumption of menses 7 months post-procedure (case level); improvement in symptoms
Tandulwadkar S. (2020) [90]	Poor responders (POSEIDON 3–4)	Pilot study; *n* = 20	Autologous bone marrow-derived stem cells + platelet-rich plasma; intraovarian instillation	Increase in AFC; increase in mature MII oocytes and secondary outcome—AMH levels (not statistically significant); and increase in numbers of Grade A and B embryos frozen on day 3	Enhanced the recruitment of dormant primordial follicles, thereby improving oocyte yield and increasing both the number and quality of embryos following controlled ovarian stimulation in POSEIDON Groups 3 and 4 poor responders.
Zafardoust S. (2023) [91]	POR	Non-randomized controlled study; *n* = 180 infertile individuals (90 treated vs. 90 control)	Menstrual blood-derived mesenchymal stromal cells; bilateral intraovarian injection	Improvement in anti-Müllerian hormone (AMH) levels (*p* = 0.0007) and antral follicle count (AFC) (*p* < 0.001) at 2 months vs. control	Higher rate of spontaneous pregnancy (*p* < 0.005) and greater numbers of mature oocytes and embryos among women who underwent ICSI/IVF
Santamaría X. (2016) [92]	Refractory Asherman’s syndrome (AS)/Endometrial atrophy (EA)	Prospective, experimental, non-controlled study; 18 patients aged 30–45 years with refractory AS or EA were recruited, and 16 of these completed the study	Autologous CD133+ BM-derived cells, delivery into the spiral arterioles by catheterization	Increase in menses volume/duration; increase in endometrial thickness; decrease in adhesion score; increase in angiogenesis process	Three patients became pregnant spontaneously, resulting in one baby boy born, one ongoing pregnancy, and a miscarriage. Furthermore, seven pregnancies were obtained after fourteen embryo transfers, resulting in three biochemical pregnancies, one miscarriage, one ectopic pregnancy, one baby born, and one ongoing pregnancy.
Cao Y. (2018) [93]	Recurrent IUA	Prospective, non-controlled, phase I clinical trial; *n* = 26	1 × 10^7^ umbilical cord-derived mesenchymal stromal cells (UC-MSCs), loaded onto a collagen scaffold, were transplanted into the uterine cavity following an adhesion separation procedure	Increase in endometrial thickness; decrease in intrauterine adhesion score; upregulation of ERα (estrogen receptor α), vimentin, Ki67, and vWF (von Willebrand factor) expression levels and downregulation of ΔNP63 expression level; DNA short tandem repeat (STR) analysis showed that the regenerated endometrium contained patient DNA only	By 30 months: 26 patients had become pregnant, and 8 of them had delivered live babies with no obvious birth defects and without placental complications, one patient in the third trimester of pregnancy, and one had a spontaneous abortion at 7 weeks
Zhang Y. (2021) [94]	Asherman syndrome	Prospective study; 18 infertile women with unresponsive thin endometrium	Collagen scaffold/umbilical cord mesenchymal stem cells transplanted into the uterine cavity in two consecutive menstrual cycles	Endometrial thickness increase from 4.08 ± 0.26 to 5.87 ± 0.77 mm (*p* < 0.001); increase in micro-vessel density, upregulated expression of Ki67, estrogen receptor alpha, and progesterone receptor	Three of fifteen patients after FET became pregnant, of whom 2 gave birth successfully and 1 had a miscarriage at 25 weeks’ gestation. One of 2 patients without FET had a natural pregnancy and gave birth normally after transplantation
Huang J. (2022) [95]	Intrauterine adhesions/cesarean scar diverticulum	Phase I; *n* = 10	Human umbilical cord MSCs twice into the uterus	Endometrial thickness, volume of the uterus, and cesarean scar diverticulum showed improving tendencies, but no significant difference was noted	Not powered for pregnancy endpoints
Singh N. (2020) [96]	Refractory AS/EA	*n* = 25	Autologous bone marrow-derived mononuclear stem cells instilled into the subendometrial zone followed by oral estrogen therapy for 3 months	Endometrial thickness increase at 3 months (3.3 to 5.1 mm), six of the seven amenorrheic patients resumed menses.	Three patients had a successful pregnancy outcome.

**Table 2:** Previous clinical studies of Potential Applications in Men: Sperm Quality Enhancement.

Study (Year)	Country	Cell Type	Patients (*n*)	Condition	Intervention	Outcomes	Limitations
Zhankina et al., 2024 [44]	Kazakhstan	Bone marrow-derived MSCs	10	Non-obstructive azoospermia (NOA)	Autologous MSC injection	Improved sperm count in 4/10 patients, partial restoration of spermatogenesis	Small sample size, short follow-up
Ismail et al., 2023 [46]	Egypt	Stem cell transplantation (animal + pilot human trial)	Preclinical + few humans	Testicular tissue injury	Testicular injection	Improved histology, some recovery of spermatogenesis	Mostly animal data, safety unclear
Modanlou et al., 2025 [49]	Review of clinical trials	MSCs, SSCs	Multiple	Male infertility (various)	Systematic analysis	Reported safety, modest improvements	Lack of large RCTs
Margiana, 2024 [50]	Indonesia	MSCs	15	NOA	Testicular injection	Some sperm retrieval, improved testicular function	Non-randomized, small cohort

Note: All studies were early phase and lacked long-term follow-up. Clinical application remains experimental.

## Data Availability

On behalf of co-authors I am submitting the enclosed paper entitled “The Use of Stem Cells in Assisted Reproduction”. “The research was based on comparison of annual reports 2010–2025”. Our profound analysis was based on comparison of annual reports from Reproduction Database and National Summary Reports presented by The Centers for Disease Control and Prevention. No new data were created or analyzed in this study.
